# Pre‐implantation genetic testing: Past, present, future

**DOI:** 10.1002/rmb2.12352

**Published:** 2020-10-13

**Authors:** Kazuhiro Takeuchi

**Affiliations:** ^1^ Takeuchi Ladies Clinic/Center for Reproductive Medicine Aira‐shi Japan

**Keywords:** fluorescence, in situ hybridization, next‐generation sequencing, PGT, TE biopsy

## Abstract

**Background:**

Pre‐implantation genetic testing (PGT) has been performed worldwide since it was first used by Handyside et al in the United Kingdom to sex embryos in 1990. Until about 2010, cleavage stage embryo biopsy and fluorescent in situ hybridization (FISH) were mainstream; however, in 2012, blastocyst biopsy (trophectoderm; TE biopsy) became mainstream. In addition, array comparative genomic hybridization (aCGH) was used for analysis and further evolved to next‐generation sequencing (NGS), which is used worldwide.

**Methods:**

PGT for reciprocal balanced translocation and Robertsonian translocation (PGT‐SR) was approved in Japan for habitual abortion to reduce pregnancy loss, and since 2008, we have been performing PGT‐SR using cleavage stage embryos and FISH. In 2014, we performed TE biopsy and NGS analysis.

**Main findings:**

In this paper, I separately described the details of our methods and clinical results of FISH and NGS. NGS is superior to FISH because it can detect all chromosomes.

**Conclusion:**

TE biopsy and NGS, which have recently become mainstream, have stable outcomes, because TE biopsy yields more cells and fewer mosaics than the cleavage stage. As a result, diagnoses are more reliable, resulting in higher pregnancy rates and lower abortion rates.

## 1 INTRODUCTION

Since PGT was introduced in assisted reproductive technology (ART), it has become a routine method for examining embryo aneuploidy and genetic disorders in embryos, worldwide. Recently, in the European Society of Human Reproduction and Embryology (ESHRE), expression pre‐implantation genetic diagnosis (PGD) and pre‐implantation genetic screening (PGS) were abbreviated as PGT; PGT was further subdivided into PGT‐M (for monogenic disorder); PGT‐SR (structural rearrangements), which causes miscarriage; and PGT‐A (aneuploidy screening). Globally, PGT‐A and PGT‐SR are indicated for translocation carriers and women of advanced maternal age to improve pregnancy rates and reduce miscarriage rates. This year, clinical research for PGT‐A in Japan began in earnest. The present manuscript discusses the history of PGT, its present status, and its future prospects.

## 2 PRE‐IMPLANTATION GENETIC TESTING: PAST

### 2.1 Testing based on blastomeres

1. PCR with cleavage stage embryos (single gene defect).
  - In 1990, Handyside et al^1^ determined the sex of embryos in patients with X‐linked recessive diseases. Disease transmission was avoided via the selection of female embryos.
  - Testing for Tay‐Sachs disease (Jones Institute for Reproductive Medicine, USA).
    The first successful PGT for Tay‐Sachs disease (intron 12) was performed in the United States in 1995.^2^
    A single blastomere was removed from a cleavage stage embryo, and its DNA was amplified by polymerase chain reaction (PCR). A restriction enzyme was then used to distinguish between affected and unaffected embryos, after which embryo transfer resulted in a healthy child.

### 2.2 Blastomere‐based detection of chromosome aberration (FISH)

1. In 1995, Munné et al^3^ at Cornell University reported that a healthy baby was born following detection of chromosome aberration using the FISH technique.
2. Following this success in the United States, PGT for recurrent pregnancy loss (balanced reciprocal translocation) was finally approved in Japan in 2008, more than 10 years later. Our clinic began clinical application of PGT in 2008, and we reported our first successful birth in 2009. Our embryo biopsy and FISH techniques are detailed below.
3. Outcomes at our clinic

#### 2.2.1 Cleavage stage embryo biopsy

Embryo biopsy poses two questions: at what stage to biopsy cells and the number of biopsies. For diagnosis, harvesting multiple cells is advantageous. In a mouse experiment, we examined which stage cells could be harvested from, and how many cells could be harvested.^4^

In both the four‐cell and eight‐cell stages, harvesting a single blastomere did not affect subsequent development into blastocysts, post‐embryo transfer implantation rates in recipients, or live birth rates (Table 1). In addition, biopsy did not affect abnormalities or deformities. The study was continued to the next generation, which also did not demonstrate any adverse effects associated with biopsy.^4^ Although blastomere removal was demonstrated not to affect subsequent development in animal experiments, does the same hold true for human embryos? With human embryos, a biopsy provides fewer cells than an animal embryo, and performing a biopsy prior to compaction is considered to result in less damage to the embryo. Therefore, the 8‐cell stage is considered suitable for embryo biopsy.^5^ We have long performed embryo biopsy primarily by extrusion, as we believe that not directly aspirating the sample blastomere results in less damage to the DNA. Our embryo biopsy procedure is described in detail below:

**TABLE 1:** Implantation and live birth rates following embryo biopsy at the 4‐cell and 8‐cell stages (Takeuchi et al^4^)

Cleavage	Groups	No. of pre‐embryos transferred	Implantation rate	Live birth rate
Four‐cell	Control	81	14/18 (77.8)^a^	42/63 (66.7)
	Biopsy			
	Enucleation	85	13/22 (59.1)	31/63 (49.2)^b^
	Aspiration	93	16/25 (64.0)	40/68 (58.8)^c^
	Extrusion	65	11/17 (64.7)	27/48 (56.3)^c^
Eight‐cell	Control	69	14/17 (82.4)	34/52 (65.4)
	Biopsy			
	Enucleation	69	10/16 (62.5)	30/53 (56.6)^c^
	Aspiration	71	10/15 (66.7)	34/56 (60.7)^c^
	Extrusion	44	10/15 (66.7)	17/29 (58.6)^c^

^a^ Values in parentheses are percentages.

^b^ *P* < .05 (enucleation versus control).

^c^ Not significantly different from the control (*P* > .05).

**FIGURE 1:**
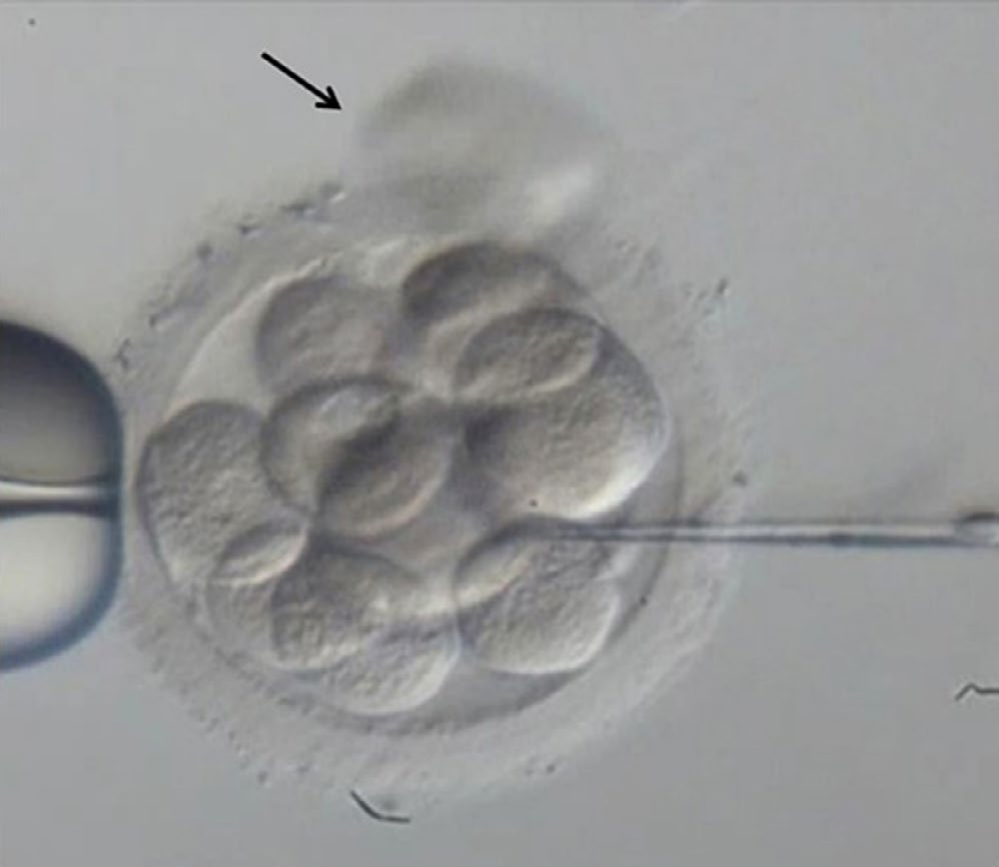
Extrusion in human embryo. A blastomere was displaced through the initial slit in the zona pellucida

*Extrusion* ^4^: This method involves using a pipette to inject culture medium into the embryo to increase internal pressure and, thus, extrude a single blastomere from the opening in the zona pellucida. First, the zona pellucida is punctured with a special needle, and the tip of the needle is hit with a holding pipette to create an opening in the zona pellucida. Next, after the culture medium is suctioned, the zona pellucida is penetrated again from the 3 o’ clock position, and the culture medium is injected into the embryo. Finally, a single blastomere is extruded from the cleavage in the zona pellucida at the 12 o’ clock position (arrow) (Figure 1).

## 3 PGT FOR BALANCED RECIPROCAL TRANSLOCATIONS USING FISH (OUTCOMES AT OUR CLINIC)

In embryo biopsies, we have long conducted PGT primarily using FISH. There are 16 possible combinations of chromosomes in gametes derived from meiosis from a reciprocal balanced translocation carrier (if the partner is normal). These combinations involve five modes of segregation: alternate segregation, adjacent‐1 segregation, adjacent‐2 segregation, 3:1 segregation, and 4:0 segregation. Alternate segregation can occur in normal people and carriers, and the phenotype of the child is normal. All other modes of segregation (unbalanced) result in abnormalities and often end in abortion. It is necessary to consider combinations of FISH signals that correspond to the combinations of chromosomes that result from fertilization of a gamete derived from meiosis.

Example of diagnosis in interphase nuclei:

The fixation of blastomeres and the actual conditions of the FISH method will be described.

1. Fixing method: Whether the FISH method is successful or not depends on the fixing method. Therefore, fixing the blastomere is one of the most important procedures. The cells obtained by biopsy were washed with PBS (‐), hypotonic treatment in 0.5% sodium citrate for 5 min, and then, Carnoy's solution (methanol: acetic acid = 3:1) was added dropwise to the slide glass. Fix on top operation under a phase‐contrast microscope, confirming that the cytoplasm was completely removed and the nucleus was attached to the slide glass. If the cytoplasm was not removed, repeat the Carnoy's solution on a glass slide.
2. Denaturation: The sample was denatured with a denaturing solution (70% formaldehyde/2xSSC × SSC) and heated to 75°C for 5 min.
3. Hybridization: Performed in a moisture box at 37°C for 12 to 16 h. Consider appropriate conditions depending on the type of probe, but if multiple probes are used, such as telomere probes, it is important to match to a probe that is difficult to obtain the signal. A signal may be obtained within a few hours, but generally it is easier to obtain a good signal for 12‐16 h (overnight).
4. Washing: After hybridization, wash with 50% formamide/ 2X SSC at 45°C, three times for 10 min, and further washed with 2X SSC for 10 min and 2X SSC/ 0.1% NP‐40 for 5 min. Next, the cells were counterstained with DAPI‐II and observed under a fluorescence microscope.

### 3.1 Select probes

The probe is divided into CEP that stains centromere, a subtelomere‐specific probe that stains telomeres at the ends, and a Locus‐specific probe that stains a specific site, depending on the portion to be stained.

The probe used, a balanced or unbalanced type, is a combination of at least three probes according to the karyotype of the balanced reciprocal translocation carrier. The balanced type is alternate segregation and becomes normal or carrier.

**FIGURE 2:**
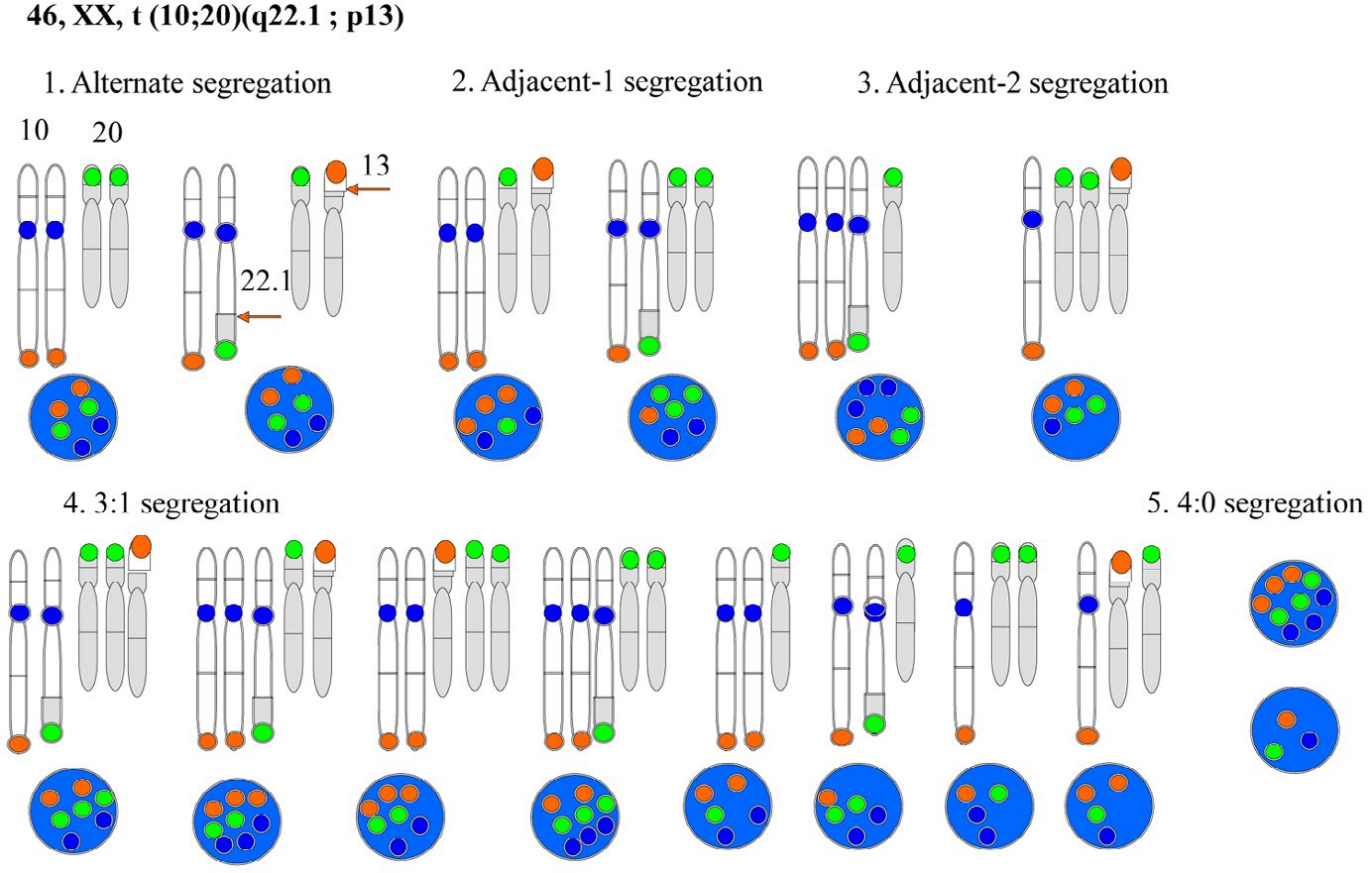
FISH staining patterns in interphase nuclei

**FIGURE 3:**
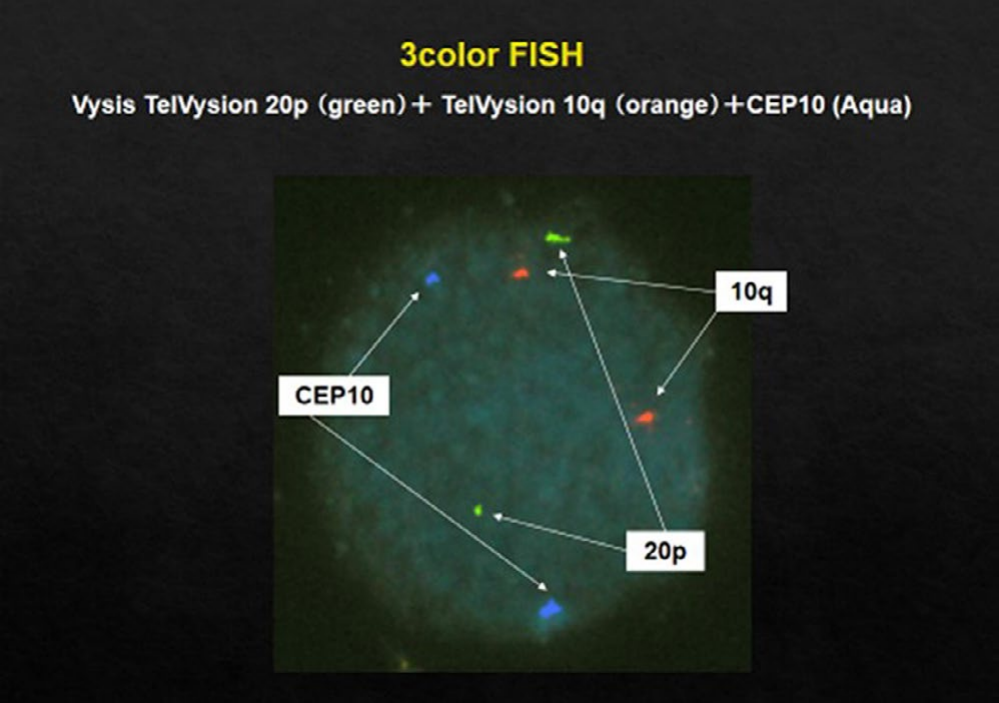
3‐color FISH

Figure 2 shows modes of segregation for fertilization between a 46, XX, t (10; 20) (q22.1; p13) carrier and a normal gamete. In this case, diagnosis was performed with three colors: CEP10 (aqua), subtelomere‐specific probe 10q (red), and subtelomere‐specific probe 20p (green). The fluorescence of these colors in a 2:2:2 ratio indicates alternate segregation and that the embryo will be normal or a carrier. All other ratios of colors indicate an unbalanced translocation. Figure 3 shows images in three color FISH of the balanced type. Table 2 shows the clinical outcomes for FISH at our clinic.

**TABLE 2:** Clinical outcomes at our clinic for PGT‐SR with FISH

Patient (Age)	No. of embryos biopsied	No. of embryos tested	No. of balanced embryos	Embryo transfer	No. of embryos transferred	Pregnancy	Course
Patient 1 (36) 46, X○, t (15; 18) (q24; q12.2)	22	20	2	(+)	1	(+)	♀ 39w + 6d C/S 3106g
Patient 2 (35) 46, X○, t (3; 15) (p13; q26.1)	15	13	2	(+)	1	(+)	IUFD 7w + 5d^a^
	15	13	2	(+)	1	(‐)	
Patient 3 (41) 46, X○, t (2;10) (p22; p15)	7	6	3	(+)	2	(‐)	
	8	8	1	(+)	1	(‐)	
Patient 4 (40) 46, X○, t (11; 22) (q23.3; q11.2)	3	2	0	(‐)			
Patient 5 (42) 46, X○, t (2; 13) (q22; q22)	11	10	2	(+)	2	(+)	♂ 39w + 6d C/S 2710g
Patient 6 (35) 46,X○,t(10;20)(q22.1;p13)	19	18	3	(+)	2	(‐)	
	Frozen‐thawed transfer of surplus blastocysts			(+)	1	(+)	♀ 36w + 0d NVD 2740g
Patient 7 (36) 46, X○, t (11; 22) (q23.3; q11.2)	21	19	5	(+)	1	(‐)	
Patient 8 (36) 45, X○, der (13; 14) (q10; q10)	13	12	5	(+)	2	(+)	♂ 37w + 5d C/S 3508g
Patient 9 (30) 45, X○, der (13; 14) (q10; q10)	17	13	3	(+)	1	(‐)	
	Frozen‐thawed transfer of surplus blastocysts following vitrification			(+)	1	(‐)	
				(+)	1	(+)	♀ 38w + 2d C/S 3114g
Patient 10 (36) 46, X○, t (6; 8) (q15; p21.1)	20	16	1	(‐)			
Patient 11 (29) 46, X○, t (4; 14) (q21.2; q21)	18	11	2	(+)	1	(+)	♂ 39w + 1d NVD 3115g
Patient 12 (40) 45, X○, der (14; 21) (q10; q10)	15	13	2	(+)	1	(+)	IUFD 8w + 6d^b^

Note

Rate of post‐PGT embryo transfer: 83.3% (10/12), pregnancy rate among transfer patients: 80.0% (8/10)

Live birth rate: 75.0% (6/8), abortion rate: 25.0% (2/8), overall pregnancy rate: 53.3% (8/15)

Abbreviations: C/S, cesarean section; IUFD, intrauterine fetal death; NVD, normal vaginal delivery.

^a^ Balanced reciprocal translocation 46, X○, t (3; 15) (q13; q26.1) in chromosomal testing of abortion product

^b^ Chromosomal testing was not conducted.

## 4 CURRENT TROPHECTODERM BIOPSY (TE BIOPSY) IN THE BLASTOCYST STAGE

For chromosomal analysis,^6^ Kokkali et al recommended performing embryo biopsy in the blastocyst stage rather than the cleavage stage, in which mosaicism is common and few cells can be obtained.^7^ Therefore, the most common method of embryo biopsy is TE biopsy.

Our TE biopsy procedure and sampling method are described below.

After preparing in advance, a dish (Falcon 351007) for the number of embryos to biopsied, a holding pipette (medion international CC), and a biopsy pipette (sunlight medical) with an inner diameter of 25 μm were installed, and then, the blastocyst was transferred to the dish. The biopsy pipette was previously washed with polyvinyl pyrrolidone (PVP) (Orgio) to prevent cell attachment, and the inside was coated. Embryos that grew into hatched blastocysts were fixed with a holding pipette, TE cells were aspirated into the biopsy pipette while avoiding inner cell mass (ICM), and laser irradiation (pulse 300 μs) (laser perforator LYKOS (Brown Technology)) was performed. The cells were collected, the obtained cell sample was discharged from a biopsy pipette, the number of cells was confirmed, and the cells were collected.

Next, the sampling will be described. As a preliminary preparation, we entered the sample number in the dish (Falcon 351007) for washing the sample cells and 0.2‐mL tube (Eppendorf PCR tubes 0.2 mL) for storing the sample.

In order to prevent cell stickiness, Pasteur, which aspirates the sample, has its tip lightly washed with PVP beforehand.

After washing the sample three times with PBS, it was moved to the bottom of the 0.2 mL tube, and the tube lid was closed.

After centrifugation, it was stored frozen (−20℃).

The actual NGS procedures are as follows: First, the whole sample subjected to biopsy was subjected to whole‐genome amplification (WGA) using a Veriseq PGS kit (Illumina) and a thermal cycler (Mastercycler Nexus, Eppendorf). The obtained sample was quantified and diluted to 0.2 ng/μL. The diluted sample was amplified with DHA tag and polymerase chain reaction (PCR).

After amplification, cleanup and normalization were performed, as well as load library, pooling, and loading. The next day, the obtained data were subjected to chart analysis using BlueFuse Multi Software (Illumina).

Thawed blastocysts were recovered using a time‐lapse incubator. For embryos in which blastocoel expansion was confirmed, TE biopsy was performed via either (a) aspiration or (b) a novel extrusion method.

### 4.1 Aspiration

**FIGURE 4:**
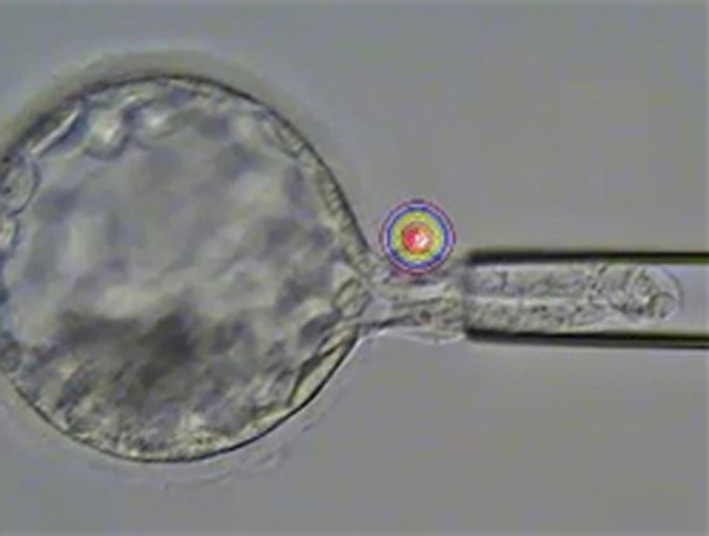
Aspiration

The LYKOS laser perforation system (Hamilton Thorne, Inc) was used at a pulse of 120 μs to create an opening in the zona pellucida of embryos for which the position of the inner cell mass can be confirmed immediately after thawing or during recovery, and which have a perivitelline space large enough to enable safe opening of the zona pellucida (Figure 4). Next, TE biopsy was performed with a biopsy pipette with an internal tip diameter of 25 µm (Sunlight Medical, Inc) and laser irradiation at a pulse of 300 µs for embryos for which the trophectoderm protrudes from the opening created as described above.

An average of 5.83 cells was harvested in each biopsy. Although the number of cells obtained in a biopsy depends greatly on the operator's technique, Kokkali et al^7^ reported a yield of 4‐5 cells, which is roughly consistent with the yield of our clinic's aspiration technique.

### 4.2 Novel extrusion method

**FIGURE 5:**
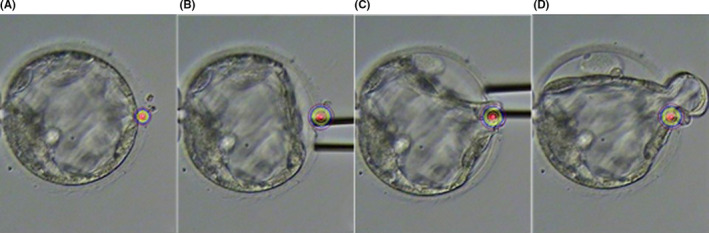
Novel extrusion method. Laser irradiation at a pulse of 120 µs was used to create a small opening in the zona pellucida. A, Culture medium was injected through the opening, and cells were extruded from the opening. B, C, Extruded cells are subjected to TE biopsy as in our aspiration method (D)

After recovery, laser irradiation at a pulse of 120 µs was used to create a small opening in the zona pellucida of embryos that developed into expanded blastocysts (Figure 5). The culture medium was injected through the opening, and cells were extruded from the opening. These extruded cells were subjected to TE biopsy, as in our aspiration method.

After TE biopsy, embryos were transferred to culture medium. After preservation of the biopsy cells was complete, they were frozen with Cryotip Vitrification (Kitazato).

A basic study at our clinic revealed no differences in post‐refreezing/rethawing recovery rates between aspiration and our novel extrusion method (Table 3).

**TABLE 3:** Recovery rates in aspiration and novel extrusion method

	No. of frozen/thawed embryos	No. of embryos recovered	Recovery rate (%)
Aspiration	18	18	100
Novel extrusion	13	13	100

**FIGURE 6:**
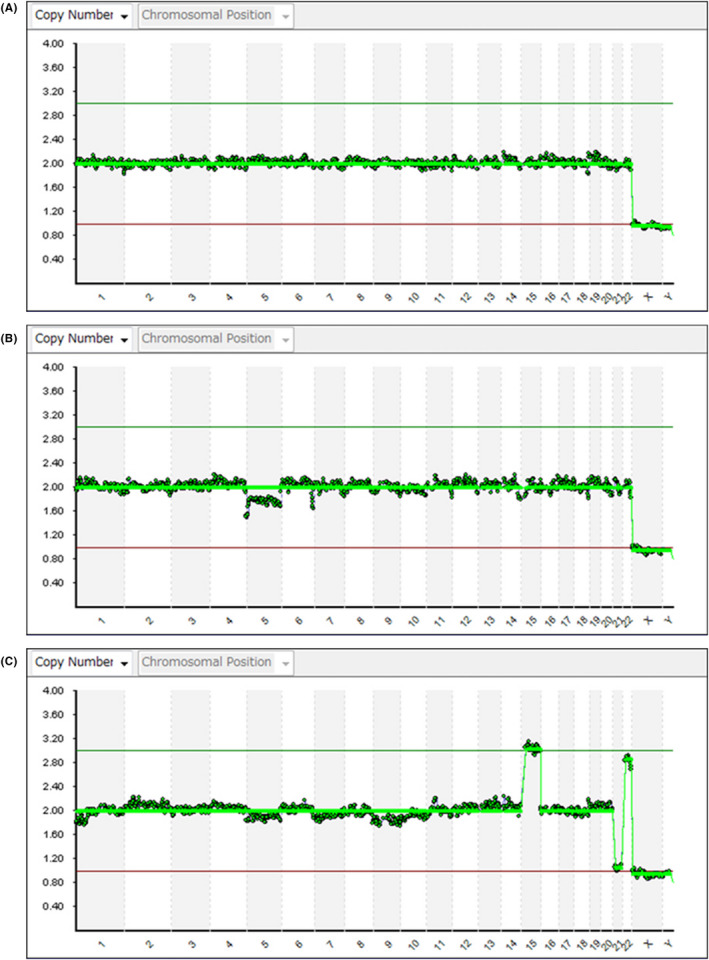
Actual NGS chart samples. A, Euploidy, B, Mosaic, C, Aneuploidy

In habitual abortion (balanced reciprocal translocation carriers, Robertsonian translocation), chromosomes are now analyzed using NGS rather than FISH. With FISH, only specific areas of chromosomes could be detected. However, NGS enables the detection of not only the translocation area but also of all chromosomes. Figure 6 shows examples of the actual charts of NGS.

Tables 4 and 5 show clinical outcomes using NGS for PGT‐SR using NGS and for PGT‐M at our clinic.

**TABLE 4:** Clinical outcomes for PGT‐SR in NGS‐based PGT at our clinic

Patient (Age)	No. of embryos biopsied	No. of embryos tested	Balance	Euploidy (Mosaicism (M))^*^	Embryo transfer	No. of embryos transferred	Pregnancy	βHCG (mIU/mL)	Outcome/remarks
Patient 1 (38) 46, X○, t (4; 18) (p15.3; q21.2)	12	12	4	1	+	1	+	536.2	♀ 40w + 1d NVD 2994g
Patient 2 (42) 46, X○, t (8; 10) (p21.3; q22.1)	10	10	5	1	+	1	+	333.4	♀ 38w + 5d C/S 2760g
Patient 3 (40) 46, X○, t (8; 19) (p21.1; q13.2) mos 46, XX [28]/47, XXX [2]	12	12	8	2	+	1	+	169	♂ 40w + 0d C/S 3678g
	Frozen‐thawed transfer of surplus blastocysts				+	1	+	614.5	Currently pregnant
Patient 4 (32) 46, X○, t (6; 11) (q23.2; q22.1)	7	7	3	1 (M 2)	+	1	+	678.2	♀ 39w + 0d NVD 2830g
Patient 5 (33) 46, X○, t (3; 13) (p23; q13)	13	12	4	1 (M 2)	+	1	‐	0.829	
	Frozen‐thawed transfer of surplus blastocysts				+	1	‐	1.22	
	Frozen‐thawed transfer of surplus blastocysts				+	1	+	384.1	♂ 39w + 6d NVD 3784g
Patient 6 (33) 46, X○, t (6; 13) (p24; q21.2)	13	13	8	2 (M 3)	+	1	+	666.7	♀ 40w + 2d NVD 3240g
Patient 7 (33) 46, X○, t (1; 13) (q12; q32)	11	11	4	3	+	1	‐	<0.001	
	Frozen‐thawed transfer of surplus blastocysts				+	1	‐	0.222	
	Frozen‐thawed transfer of surplus blastocysts				+	1	+	69.95	♀ 41w + 4d NVD 2696g
Patient 8 (37) 46, X○, t (2; 8) (q12; q24.22)	10	10	4	1 (M 1)	+	1	‐	0.107	
	Frozen‐thawed transfer of surplus blastocysts				+	1	‐	<0.500	
Patient 9 (34) 46, X, ?inv (Y) (p11.2q11.2), t (12; 13) (p12.3; q21.1)	5	5	3	1 (M 1)	‐				♀ 39w + 4d NVD 3250g
Patient 10 (34) 45, X○, der (13; 14) (q10; q10)	9	9	8	4 (M 2)	+	1	‐	386.2	GS‐ Chemical abortion
	Frozen‐thawed transfer of surplus blastocysts				+	1	‐	14.7	GS‐ Chemical abortion
Patient 11 (42) 46, X○, t (11; 22) (q23.3; q11.2)	5	5	1	0 (M 1)	+	1	‐	55.86	GS‐ Chemical abortion
Patient 12 (39) 46, X○, ins (2; 7) (q31; p11.2p13)	11	11	6	3	+	1	+	1208	♂ 38w + 0d C/S 2648g
Patient 13 (36) 46 X○, t (2; 5) (q21.3; q33.3)	4	4	1	1 (M)	+	1	+	357.5	♂ 39w + 0d NVD 3354g
Patient 14 (30) 46, X○, t (4; 8) (q33; q21.1)	7	7	1	0	‐				
	5	5	0	0	‐				

Note

GS, Gestational Sac.

**TABLE 5:** Clinical outcomes in PGT‐M at our clinic

SMA									
Patient (Age)	No. of embryos biopsied	No. of embryos tested	No. of embryos unaffected	No. of embryos affected	Embryo transfer	No. of embryos transferred	Pregnancy	βHCG (mIU/mL)	Outcome/remarks
Patient 1 (38)	11	11	8	2	＋	1	＋	237.5	♀ 40w + 2d NVD 3036g
Patient 2 (40)	8	8	4	0	＋	1	＋	902.1	Currently pregnant

DMI								
Patient (Age)	No. of embryos biopsied	No. of embryos tested	No. of embryos unaffected	Embryo transfer	No. of embryos transferred	Pregnancy	βHCG (mIU/mL)	Outcome/remarks
Patient 1 (42)	5	5	3	－				

## 5 WHAT IS THE RIGHT CELL STAGE FOR THE EMBRYO BIOPSY?

Polar bodies have long been used for analysis. Although they offer the advantages of minimal invasiveness and lack of mosaics in chromosomal analysis, they only yield maternal information and are therefore not frequently used today. Next, cleavage stage embryos, which have been used in analysis longer than embryos of any other stage.

As described earlier, we have long used embryos at the 8‐cell stage in the diagnosis of balanced reciprocal translocation with FISH. In a normal diagnosis, taking a single blastomere from an 8‐cell embryo is considered sufficient.

However, in comparisons of day 3 biopsy and blastocyst biopsy, Kokkali et al^7^ found a significantly higher implantation rate with blastocyst biopsy. In a day 3 biopsy, biopsy embryos were selected based on morphological assessment. This selection method involves handling more embryos than in a blastocyst biopsy, which targets only embryos that developed into blastocysts. Consequently, a day 3 biopsy requires greater time and effort. In addition, in a blastocyst biopsy, analysis takes more than 24 hours, thus requiring the embryo to be frozen. However, in our experiment, post‐biopsy embryo survival rates were similar to those of normal embryos, the recovery rate was high, and freezing did not pose any problems.

One problem that has been raised in PGT is the potential for misdiagnosis due to mosaic embryos.^8^ While some studies have stated that, the trophectoderms of blastocysts are often present with mosaicism, Magli et al^9^ and Josien et al^10^ hold that the status of aneuploidy and other forms of mosaicism does not change in the inner cell mass or trophectoderms of a blastocyst. This assertion suggests that the entire embryo can be diagnosed by harvesting trophectoderms.

The greatest advantage of blastocyst biopsy is the ability to harvest a larger numbers of cells. In aCGH and NGS, which are conducted to analyze anomalies in the primary structure of the genome, analysis of a single blastomere remains difficult because of the small amount of template DNA. However, blastocyst biopsy is now the dominant method because it yields more cells, and can be detected as mosaics. Table 6 shows the advantages and disadvantages of biopsy in each embryonic stage.

**TABLE 6:** Advantages and disadvantages of biopsy in each embryonic stage

	Advantages	Disadvantages
Polar body	No effect on embryogenesis Minimally invasive, no mosaics	Provides only maternal information
Cleavage stage	Historically the most common Large nuclei well suited to FISH	Yields only a single cell Many mosaics
Blastocyst (TE biopsy)	Yields many specimens (≥5 cells) Few mosaics	Requires technical training
Blastocoelic fluid	Minimal damage to embryo Simple technique	Little replication study data Pervasive negative opinion
Spent media	Simple Biopsy not required	Little replication study data *Further study needed for clinical application*

## 6 THE FUTURE OF PRE‐IMPLANTATION GENETIC TESTING (STRIDES TOWARD MINIMAL INVASIVENESS)

### 6.1 Blastocoelic fluid aspiration^11^

Blastocoelic fluid aspiration was first reported by Magli et al,^11^ who reported the following advantages: (a) it involves little invasion into the embryo; (b) it does not involve cell collection, and is therefore ethically acceptable; (c) blastocoelic fluid expands sometime after collection, and can thus be collected multiple times; (d) concordance with polar bodies, blastomeres, and TE biopsy was very high. In addition, Zhang et al^12^ reported that in PGT conducted with blastocoelic fluid, NGS had an efficiency of 84%, meaning that blastocoelic fluid is useful as a source of DNA.

However, in a negative opinion, Capalbo et al^13^ reported that blastocoelic fluid analysis showed a high amplification failure rate (65%), and that even when amplification was successful, the concordance rate with TE was only 37.5%. In addition, Tobler et al^14^ found a high discordance of 52% between blastocoelic fluid and inner cell mass‐TE, leading them to conclude that blastocoelic fluid is not well suited for clinical application. The above findings suggest that further study is required before blastocoelic fluid can be clinically applied to PGT.

### 6.2 Spent media

**FIGURE 7:**
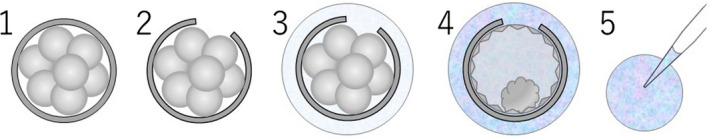
Procedure for collecting spent embryo culture media. 1. Transfer embryos from the culture medium on day 3 (G1). 2. Perform assisted hatching (AH) with laser on day 3. 3. Culture embryos from day 3 to day 5 (6). 4. Perform TE biopsy. 5. Collect the spent medium and cryopreserve until analysis (*Shamonki MI, et al Proof of concept: pre‐implantation genetic screening without embryo biopsy through analysis of cell‐free DNA in spent embryo culture media. Fertil Steril 2016*)

Several studies have recently attempted to conduct PGT using spent embryo culture media.^15^, ^16^, ^17^, ^18^, ^19^ Figure 7 shows the general procedure for collecting the spent embryo culture media. While blastocoelic fluid collection is also minimally invasive, spent media analysis does not require biopsy of the embryo itself, and anyone can collect spent media. Thus, spent media analysis is likely to be studied further in the future. In spent media analysis, the greatest concern is contamination of maternal cells.

In the analysis of medium used to culture embryos from day 3 to 5, Lane et al^17^ observed a low concordance with TE biopsy, which they considered the result of contamination of maternal cells (granulosa cells).

In a recent study, Rubio et al^18^ reported that embryo free DNA analysis was 78.2% concordant with the corresponding TE biopsy, and no significant differences were detected among multiple centers ranging from 72.5% to 86.3%.

Moreover, they reported that concordance rates exceeded 86% when all defined steps in the culture laboratory were controlled to minimize the impact of maternal and operator contamination.

Ho et al^19^ compared concordance with TE biopsy for ploidy and sex in two groups of embryos: one that underwent assisted hatching (AH) on days 3 and 5, and one that did not undergo AH (Table 7).

**TABLE 7:** Concordance of spent medium cfDNA with TE biopsy based on use/non‐use of AH (Ho et al^19^)

Pair	Concordance for ploidy			*P* value^a^	Concordance for sex			*P* value^a^
	Total	AH	No AH		Total	AH	No AH	
Day 3 cf DNA vs, whole embryo^b^ (*n* = 16)	9/16 (56.3%)	4/8 (50.0%)	5/8 (62.5%)	.61	13/16 (81.3%)	5/8 (62.5%)	8/8 (100%)	.06
Day 5 cf DNA vs, whole embryo^b^ (*n* = 33)	15/33 (45.5%)	5/16 (31.3%)	10/17 (58.8%)	.11	26/33 (78.7%)	12/16 (75.0%)	14/17 (82.4%)	.61
Day 5 cDNA vs, trophectoderm biopsy^c^ (*n* = 40)	26/40 (65.0%)	16/28 (57.1%)	10/12 (83.3%)	.16	28/40 (70.0%)	17/28 (60.7%)	11/12 (91.7%)	.07
Day 5 trophectoderm biopsy vs, whole embryo^b^ (*n* = 27)	25/27 (92.6%)	12/14 (85.7%)	13/13 (100%)	.22	26/27 (96.3%)	14/14 (100%)	12/13 (92.3%)	.48

Note

Concordance rates between cell‐free DNA (cfDNA), trophectoderm biopsy, and whole embryos, *n* (%)

AH, assisted hatching.

^a^ Chi‐square analysis or Fisher exact test was used to compare AH vs. no AH groups; *P *< .05 was considered significant.

^b^ Includes research embryos only.

^c^ Includes both research embryos and clinical samples.

Concordance was found to be higher on day 5 than on day 3, while embryos that did not undergo AH were surprisingly found to exhibit a higher concordance. Although AH on day 3 was generally considered necessary in spent medium analysis, Ho et al^19^ reported that not only is AH not necessary, not performing AH, in fact, yields results that are more favorable.

## 7 CURRENT STATE OF PGT OUTSIDE JAPAN AND FUTURE ISSUES

Table 8 shows aggregated PGD/PGS data collected by the ESHRE PGD Consortium up to 2012.^20^ While publicly available reports unfortunately currently only cover up to 2012, according to the ESHRE statistics, as of 2012, PGT has been performed 45 163 cycles worldwide, PGT (SR, M) has 17 721 cycles, and PGT‐A has 26 737 cycles. At present, most of the embryo biopsies in the splitting period account for approximately 70% of the total, and the diagnostic method is FISH or PCR. After that, blastocyst biopsy and NGS were more commonly used, and according to the PGT consortium report held in Vienna in 2019, from 2017 to 2019, according to the statistics from 52 facilities worldwide, there were 4012 cases of PGT; comprising; PGT‐A, 40%, PGT‐M 38%, PGT‐SR 16%, and the other 6%, but there are many cases where PGT‐M was performed at the same time.

**TABLE 8:** Overall cycle data collection (current state of PGT outside Japan) (De Rycke et al^20^)

Indication	PGD^a^	PGS	Total^b^
Cycles to OR	17 721	26 737	45 163
Number infertile	6102	21 420	27 633
Cancelled before IVF/ICSI	53	3	56
ART method			
IVF	1672	2843	4684
ICSI	15 730	23 348	39 584
IVF + ICSI	89	404	499
Frozen + ICSI +IVF + unknown	189	89	302
Unknown	24	51	75
Cancelled after IVF/ICSI	759	481	1257
Cycles to PGS/PGD	16 945	26 254	43 887
Analysis method			
FISH	7840	26 093	34 439
PCR	8712	10	8904
FISH + PCR	93	0	93
PCR + WGA	196	0	196
FISH + PCR +WGA	2	0	2
Arrays	63	127	190
FISH + arrays	0	4	4
WGA + arrays	2	15	17
Zona breaching			
AT drilling	5332	6493	11 851
Laser drilling	10 643	17 370	28 248
Mechanical	956	2326	3709
Unknown	14	65	79
Biopsy method			
PB biopsy	329	5239	5568
Cleavage aspiration	15 726	19 821	35 728
Cleavage extrusion	576	1005	2087
Cleavage flow displacement	16	22	38
Blastocyst	142	54	197
PB and cleavage	82	26	108
Unknown	16	52	68
Embryology			
COCs	234 850	300 194	544 803
Inseminated	197 272	248 433	453 851
Fertilized	139 790	173 325	318 828
Biopsied	108 478	141 722	254 725
Successfully biopsied	106 980	140 523	251 885
Diagnosed	97 497	131 267	232 691
Transferable	36 107	45 090	82 730
Transferred	22 121	33 335	56 491
Frozen	6191	6090	12 650
Clinical outcome			
Cycles to ET	12 785	19 117	32 420
hCG positive	4742	6768	11 713
Positive heartbeat	3755	5350	9253
Clinical pregnancy rate (% per OR/% per ET)	21/29	20/28	20/29

Abbreviations: AT, acid Tyrode's; COC, cumulus oocyte complexes; ET, embryo transfer; OR, oocyte retrieval; PB, polar body; SS, social sexing; WGA, whole‐genome amplification.

^a^ PGD column includes PGD for chromosome abnormalities, sexing for X‐linked disease, and PGD for single gene disorders.

^b^ Total includes PGD and PGS for data I–XIII, as well as social sexing cycles for data I–XII (705 cycles). From data XIII onwards, details of social sexing cycles were no longer reported.

It is also worth noting that the breakdown of PGT‐M includes hereditary cancers, such as breast cancer.

The biopsy time was 40% in the blastocyst stage, and in cases of PGT‐M, there are still cleavage stage embryos and polar body biopsies, but PGT‐A is mostly in the blastocyst stage, and analysis is mainly NGS. It is noteworthy that, outside Japan, PGT‐A is performed more commonly than PGT‐SR and PGT‐M. In the past several years, PGT‐A has become particularly common. According to “ESHRE PGT Consortium good practice recommendation^21^” cited indication for PGT‐A included for PGT‐A: (a) advanced maternal age, (b) recurrent miscarriage, (c) recurrent IVF failure, (d) oocyte donation, (e) non‐medical indication, and (f) severe male factor infertility.

Outside Japan, PGT‐A is common. Although opinions regarding PGT‐A have undeniably been divided in recent years, many recent studies reported that PGT‐A is effective. In one such study, Schoolcraft et al^22^ stated that PGT‐A yielded a significantly increased implantation rate and fetal heartbeat positive rate.

In chromosomal screening with aCGH, Fragouli et al^23^ stated that blastocyst biopsy (trophectoderm analysis) is a potent method for accurately detecting numerical chromosome abnormalities, and that most mosaic blastocysts contained no normal cells. While Fragouli et al stated that mosaic embryos should not be selected for embryo transfer to begin with, some believe that many mosaic embryos result in normal births.

At any rate, blastocyst biopsy appears to be necessary to obtain large numbers of cells for PGT‐A. Some aspects of minimally invasive embryo biopsy may still need to be examined before it can be completely applied in clinical practice. In the field of assisted reproductive technology, further study may be necessary to determine whether PGT‐A is truly effective for obtaining live births.

## DISCLOSURES

I, Kazuhiro Takeuchi, declare that I have no conflicts of interest.

Human rights statement and informed consent: All procedures followed were in accordance with the ethical standards of the responsible committee on human experimentation (institutional and national), and with the Helsinki Declaration of 1964 and its later amendments. Informed consent was obtained from all patients in this study.

Approval by the ethics committee: All procedures that involved human participants were carried out in accordance with the ethical standards of the institutional review board of the Takeuchi ladies clinic.
